# Components of the ubiquitin-proteasome pathway compete for surfaces on Rad23 family proteins

**DOI:** 10.1186/1471-2091-9-4

**Published:** 2008-01-30

**Authors:** Amanda M Goh, Kylie J Walters, Suzanne Elsasser, Rati Verma, Raymond J Deshaies, Daniel Finley, Peter M Howley

**Affiliations:** 1 Department of Pathology, Harvard Medical School, Boston, Massachusetts, USA; 2 Department of Biochemistry, Molecular Biology and Biophysics, University of Minnesota, Minneapolis, Minnesota, USA; 3 Department of Cell Biology, Harvard Medical School, Boston, Massachusetts, USA; 4 Department of Biology, Howard Hughes Medical Institute, California Institute of Technology, Pasadena, California, USA; 5 Institute of Molecular and Cell Biology, Singapore

## Abstract

**Background:**

The delivery of ubiquitinated proteins to the proteasome for degradation is a key step in the regulation of the ubiquitin-proteasome pathway, yet the mechanisms underlying this step are not understood in detail. The Rad23 family of proteins is known to bind ubiquitinated proteins through its two ubiquitin-associated (UBA) domains, and may participate in the delivery of ubiquitinated proteins to the proteasome through docking via the Rad23 ubiquitin-like (UBL) domain.

**Results:**

In this study, we investigate how the interaction between the UBL and UBA domains may modulate ubiquitin recognition and the delivery of ubiquitinated proteins to the proteasome by autoinhibition. We have explored a competitive binding model using specific mutations in the UBL domain. Disrupting the intramolecular UBL-UBA domain interactions in HHR23A indeed potentiates ubiquitin-binding. Additionally, the analogous surface on the Rad23 UBL domain overlaps with that required for interaction with both proteasomes and the ubiquitin ligase Ufd2. We have found that mutation of residues on this surface affects the ability of Rad23 to deliver ubiquitinated proteins to the proteasome.

**Conclusion:**

We conclude that the competition of ubiquitin-proteasome pathway components for surfaces on Rad23 is important for the role of the Rad23 family proteins in proteasomal targeting.

## Background

Targeted protein degradation by the ubiquitin-proteasome pathway is a key means of regulating a wide variety of cellular processes, ranging from cell cycle progression [1] to antigen presentation [2]. In this pathway, an enzymatic cascade covalently attaches ubiquitin to a lysine residue on substrate proteins. The subsequent conjugation of more ubiquitin moieties, each typically linked through Lys48 of ubiquitin, results in a polyubiquitin chain that directs substrate proteins to the proteasome where they are degraded (reviewed in [3]). The importance of this proteasome-targeting step was demonstrated by experiments showing that the artificial localization of proteins to the proteasome is sufficient to cause their degradation [4].

Recent work indicates that ubiquitin receptors, which bind ubiquitin but are not intrinsic subunits of the proteasome, facilitate the docking of ubiquitinated substrates to the proteasome [5-8]. The best-studied of these receptors are the UBL-UBA proteins and include three groups: Rad23/HHR23A/HHR23B, Dsk2/PLIC1/PLIC2, and Ddi1. Rad23, for example, has been shown to play a role in the targeting of the cyclin-dependent kinase inhibitor Sic1 to the proteasome [5], and Ddi1 mediates degradation of the Ho endonuclease [9] and of the F-box protein Ufo1 [10].

Each Rad23 family member has a ubiquitin-like (UBL) domain that binds proteasomes [11-13] as well as two ubiquitin-associated (UBA) domains that bind ubiquitin [14-17]. The structure of HHR23A has been solved by NMR spectroscopy [18,19], which revealed that the UBL and UBA domains interact intramolecularly in a highly dynamic manner, as each UBA domain competes for an overlapping UBL domain surface [18]. The UBL domain of HHR23A has also been shown to bind to proteasomal subunit S5a [12], and notably, the UBL surface bound by S5a overlaps significantly with that bound by the UBA domains [18]. As with the UBL domain of HHR23A, the UBA domains also have multifunctional surfaces: specifically, the UBL- and ubiquitin-binding surfaces overlap. The binding of S5a or ubiquitin to HHR23A thus disrupts the intramolecular UBL-UBA interactions and drives HHR23A into an open conformation [18,20]. We hypothesize that these conformational changes, governed by UBL-UBA interactions, are important for HHR23A function.

To determine how UBL and UBA domain interactions contribute to Rad23/HHR23A function in ubiquitin-mediated proteolysis, we identified mutations that disrupt UBL-UBA binding, then tested the ability of the mutant proteins to bind components of the ubiquitin-proteasome pathway and to mediate delivery of a ubiquitinated substrate to the proteasome. Our results show that the interactions of the UBL and UBA domains with each other and with other proteins are interdependent, and that modulating proteasome-binding is important for the role of Rad23/HHR23 in proteasomal targeting.

## Results

### Identification of UBL mutations that reduce UBA-binding

To identify UBL mutations that affect UBA-binding, we established an affinity column chromatography assay using resin-bound UBA domains and mobile ligands. To demonstrate that our assay can distinguish proteins based on their relative affinities for the UBA domains, control experiments were performed with ubiquitin and SUMO. Both proteins are similar in size and structure to the UBL domain but ubiquitin binds the UBA domains whereas SUMO does not [14,15,21]. Equal amounts of hemagglutinin-tagged SUMO (HA-SUMO), polyhistidine-tagged ubiquitin (Ub-His), and FLAG-tagged wild-type HHR23A UBL (UBL-FLAG) were mixed and loaded onto the GST-HHR23A~ΔUBL column. HA-SUMO eluted from the column first, followed by the wild-type UBL. Ub-His eluted from the column only when the salt concentration in the running buffer was increased from 150 mM to 400 mM (Additional file 1A). Therefore, the order in which ubiquitin, UBL and SUMO eluted from the GST-HHR23A~ΔUBL column corresponded to their relative ability to interact with UBA domains. To confirm that the difference in retention time of each protein on the column is specifically due to their respective abilities to bind the UBA domains, we conducted a similar control experiment using a glutathione-sepharose column charged with GST only. HA-SUMO, Ub-His and UBL-FLAG all eluted from the GST column simultaneously (Additional file 1B).

**Figure 1:**
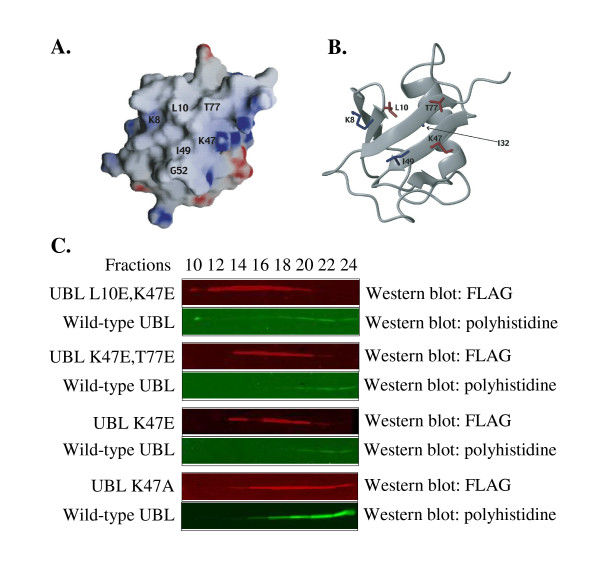
**Glutamic acid mutations in the UBL domain reduce binding to the UBA domains**. (A) The electrostatic potential is mapped onto the surface diagram of the UBL domain of HHR23A. Positive and negative charges are indicated by blue and red respectively. GRASP was used to generate the surface diagram, using the parameters -25.4 to -12.7 and 25.4 to 12.7 kT to generate the surface potentials. (B) The ribbon diagram of the UBL domain of HHR23A was generated with MOLMOL [37], using reported UBL domain coordinates for HHR23A. (C) Equal amounts of wild-type UBL-His and mutated UBL-FLAG were loaded onto a GST-HHR23A~ΔUBL column. The column was developed with PBS and fractions were collected for analysis by Western blotting, using antibodies against each epitope tag. Visualization of the proteins was performed with LI-COR's Odyssey Imaging System.

To abrogate UBL-UBA binding, we mutated residues located on the UBA-binding surface of HHR23A [18] that are conserved in the other human homolog of Rad23, HHR23B. We also considered data from previous structural studies of HHR23A, which showed that UBL-UBA binding is mediated mainly by hydrophobic interactions and that specificity is conferred by the topology of the binding surfaces [18,22]. The UBA-binding surface of the UBL domain is predominantly hydrophobic with a few basic regions [23] whereas the UBL-binding surface of the UBA domains are similarly hydrophobic but with a few acidic residues [20]. Glutamic acid mutations in the UBA-binding surface would cause electrostatic repulsion against the UBA domains and thus be effective at disrupting the UBL-UBA interaction. Therefore, we mutated L10, K47 and T77 of HHR23A to glutamic acid, individually or in combination (as indicated in Figure 1A).

To determine the affinity of these UBL mutants for UBA domains relative to wild-type UBL, UBL domain constructs were mutated accordingly and tested in our affinity column chromatography assay. As shown in Figure 1C, wild-type UBL began eluting off the column in fractions 18–20 and the elution profile of the K47A UBL mutant is identical to that of the wild-type UBL domain. In contrast, the L10E/K47E double mutant began to elute in fraction 12 and the K47/T77E and K47E mutants in fraction 14. These data indicate that the glutamic acid UBL mutants, but not the alanine mutant, have a reduced ability to bind the UBA domains relative to the wild-type UBL.

### HHR23A bearing UBA-binding mutations in the UBL domain show enhanced binding to polyubiquitin

**Figure 2:**
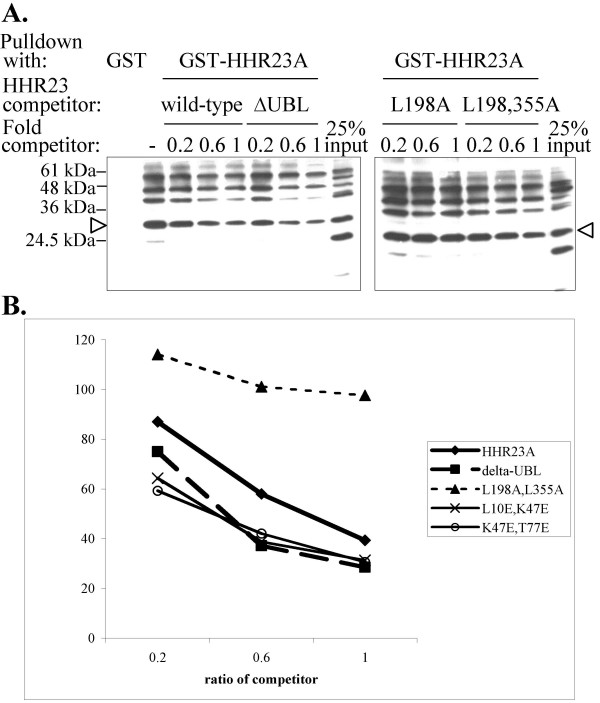
**HHR23A mutations that disrupt the UBL-UBA interaction enhance ubiquitin-binding**. (A) GST-HHR23A, untagged mutant HHR23A, free polyubiquitin chains, and glutathione-sepharose beads were mixed together. After the resin was washed, the bound proteins were eluted and resolved by SDS-PAGE and Western blotting performed with an antibody against polyubiquitin. While all the bands are conjugates of free ubiquitin, the arrow indicates that which corresponds to tetraubiquitin. (B) The band corresponding to tetraubiquitin was quantified with a Biorad Fluor-S Max phosphoimager and data from a representative experiment are presented here.

The ubiquitin-binding surface on the UBA domains overlaps significantly with that involved in binding the UBL domain [19,20]. Disrupting the intramolecular UBL-UBA domain interactions is expected to make the UBA domains more accessible to ubiquitin. We probed the affinity of mutant HHR23A protein for ubiquitin binding by assessing the extent to which each competed with resin-bound GST-HHR23A for polyubiquitin-binding. As shown in Figure 2A, polyubiquitin bound GST-HHR23A but not GST alone. As increasing amounts of untagged wild-type HHR23A was used as a competitor, the amount of polyubiquitin bound to GST-HHR23A decreased. As expected, HHR23A~ΔUBL competed with GST-HHR23A more effectively than wild-type HHR23A for polyubiquitin-binding whereas the HHR23A L198A/L355A double mutant, in which the UBA domains are unfolded and thus unable to bind ubiquitin [14,20], did not compete at all.

The HHR23A UBL mutants were then tested as competitors in the GST-pulldown competition assay. All the bands in the polyubiquitin Western blot are conjugates of free ubiquitin, but rather than quantify a smear, we quantified the specific band corresponding to tetra-ubiquitin, which is the minimum length required for proteasomal targeting [24]. The amount of tetra-ubiquitin bound to GST-HHR23A in the presence of competitor was then expressed as a percentage of the amount of tetra-ubiquitin bound in the absence of competitor (Figure 2B). When wild-type HHR23A was used as a competitor, the amount of polyubiquitin bound to GST-HHR23A decreased from 87% to 39% as the ratio of competitor to GST-HHR23A was increased from 0.2 to 1. As expected, HHR23A~ΔUBL was a more effective competitor than wild-type HHR23A, with only 75% of ubiquitin bound at a competitor to GST-HHR23A ratio of 0.2, and only 29% bound at a ratio of 1. This result is consistent with previous findings that HHR23A lacking the UBL domain exhibits higher affinity for polyubiquitin chains compared to the full-length protein [25].

In contrast, the UBA domain double mutant L198A/L355A did not compete with GST-HHR23A for polyubiquitin binding. The UBL domain mutants L10E/K47E and K47E/T77E, which showed reduced UBA-binding (Figure 1C), competed for ubiquitin-binding more effectively than wild-type HHR23A, and to a similar extent as HHR23A~ΔUBL. Our results demonstrate that UBL-UBA domain interactions reduce HHR23A's ability to bind ubiquitin. They also provide evidence that the enhanced polyubiquitin-binding of HHR23A~ΔUBL relative to wild-type HHR23A is due to loss of auto-inhibition mediated by UBL-UBA domain interactions.

### UBA-binding mutations in the Rad23 UBL domain impair proteasome-binding

In addition to regulating ubiquitin-binding, UBL-UBA domain interactions could also affect proteasome-binding such that it is enhanced when the HHR23 proteins are bound to ubiquitinated proteins. For this hypothesis to be true, the proteasome must bind a surface on the UBL domain overlapping with that which binds the UBA domains. Indeed, the UBL domain is necessary and sufficient for interaction with the proteasome [11] and the binding of HHR23A to S5a disrupts UBL-UBA domain binding [18]. Therefore, we tested whether the UBA-binding surface of HHR23 family proteins is required for proteasome interaction.

Just as ubiquitin itself binds multiple subunits of the proteasome, including Rpn10/S5a and Rpt6/S6' [26,27], so could the Rad23/HHR23 proteins. Therefore, we tested the ability of our UBL mutants, which are defective in UBA-binding, to interact with purified proteasomes instead of with specific subunits. We also used the budding yeast *Saccharomyces cerevisiae *as a model system as it is better-characterized and easier to manipulate genetically.

**Figure 3:**
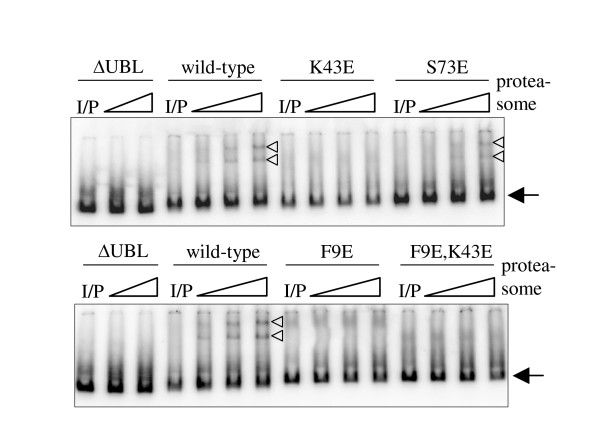
**Rad23 UBL mutants show no detectable interaction with the proteasome *in vitro***. All Rad23 constructs were bacterially expressed, purified and radiolabelled with [γ-^32^P]ATP *in vitro *via an N-terminal HMK site. After quantification by scintillation counting and by Bradford assay, the radiolabelled proteins were normalized by counts and mixed with purified yeast proteasomes in a 1:10, 1:20 and 1:50 molar excess of proteasome over proteins. After incubation at 30°C for 15 minutes, the mixtures were electrophoresed on a 3.5% native gel at 100 V for 5 hours at 4°C. The arrows indicate free Rad23 proteins and the triangles indicate bands corresponding to proteasome-bound Rad23 proteins. "I/P" denotes the lane containing the input radiolabelled protein without any proteasome.

L10, K47 and T77 in HHR23A correspond to F9, K43 and S73 in Rad23 respectively, as determined by sequence alignment. We mutated these Rad23 residues to glutamic acid as we had done for HHR23A. To test proteasome-binding, we used recombinant, purified ^32^P-labelled Rad23 proteins and purified yeast proteasomes in a electrophoretic mobility shift assay. In the presence of proteasomes, the mobility of wild-type Rad23, but not Rad23~ΔUBL, decreased, as indicated by the arrowheads in Figure 3. The intensity of the shifted band is an indicator of the amount of proteasome-bound Rad23, which increased as more proteasome was titrated into the reaction. With the exception of the S73E mutant, the Rad23 UBL mutants showed no detectable interaction with the proteasome *in vitro*. This finding provides strong evidence that Rad23 binds the proteasome via the same surface that is responsible for S5a- and UBA-binding in HHR23A.

### Mutation of the Rad23 UBL domain impairs interactions with Ufd2

**Figure 4:**
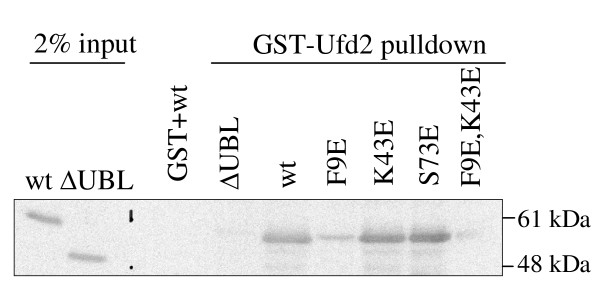
**Mutation of Rad23 F9 reduces interaction with Ufd2**. GST-pulldown experiments were performed by mixing each purified Rad23 protein with GST-Ufd2 bound to glutathione-sepharose beads. After mixing at 4°C for 1 hour, the resin was washed, and bound proteins were eluted and analyzed by SDS-PAGE and Western blotting for Rad23.

In addition to the proteasome, the Rad23/HHR23A UBL domain binds other proteins involved in ubiquitin-mediated degradation, including the ubiquitin ligase Ufd2 [6,28]. We thus tested whether our UBL mutants retain their ability to interact with Ufd2. We used bacterially expressed and purified GST-Ufd2 and Rad23 proteins in GST-pulldown experiments, following which the bound Rad23 proteins were detected by Western blotting for Rad23. As expected, wild-type Rad23 bound to GST-Ufd2 but not to GST alone, whereas Rad23ΔUBL did not bind to GST-Ufd2 (Figure 4). Interestingly, Ufd2-binding was significantly reduced by the mutation of F9, but not of K43 or of S73. This finding suggests that hydrophobic interactions govern Ufd2 binding to Rad23, and that the surfaces of Rad23 bound by Ufd2 and the proteasome do indeed overlap partially.

### UBA-binding mutations in the Rad23 UBL domain impair recruitment of a model substrate to the proteasome

The degradation of ubiquitinated substrates by proteasomes is tightly coupled to their deubiquitination by the metalloisopeptidase activity of the Rpn11 subunit [29,30]. The deubiquitinating activity of Rpn11 can be unmasked *in vitro *in the presence of a 20S proteolytic inhibitor such as epoxomicin, and can be used as a functional readout for recruitment of model ubiquitinated substrates to purified 26S proteasomes [31]. Therefore, we used the CDK inhibitor Sic1 as a substrate in an in vitro deubiquitination assay to determine the ability of each Rad23 UBL mutant to target ubiquitinated substrates to the proteasome.

**Figure 5:**
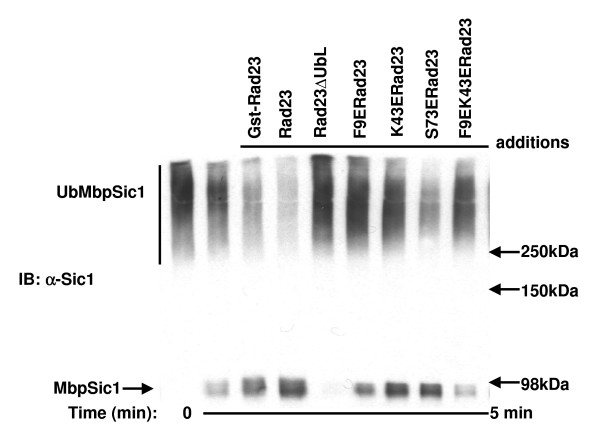
**Mutation of the Rad23 UBL domain impairs proteasomal targeting**. Recombinant Sic1 was ubiquitinated *in vitro*, proteasomes lacking Rad23 were treated with epoxomycin, and the two were then incubated at 30°C in the absence or presence of purified Rad23 proteins for 45 minutes. Deubiquitination of Sic1 was then analyzed by Western blotting for Sic1.

In this assay, ubiquitinated MbpSic1 is prepared with SCF complex (Figure 5 lane 1, which represents 100% pre-formed ubiquitinated substrate). Upon the addition of Rad23-deficient purified proteasomes, the ubiquitinated MbpSic1 is targeted to the proteasome and deubiquitinated. Prior treatment of the proteasomes with epoxomicin inhibits substrate degradation and the extent to which deubiquitination occurs may be assessed by Western blotting for Sic1.

As shown in Figure 5, the proteasome exhibited basal deubiquitinating activity in the absence of wild-type Rad23. Sic1 deubiquitination increased in the presence of wild-type Rad23 (lanes 3 and 4), most likely due to the ability of Rad23 to facilitate delivery of ubiquitinated Sic1 to the proteasome. Sic1 deubiquitination was blocked by Rad23~ΔUBL (lane 5) and limited to the basal level in the presence of the F9E/K43E mutant, which showed no detectable binding to the proteasome (Figure 3). In contrast, Sic1 deubiquitination was enhanced by the S73E mutant, which retained proteasome-binding (Figure 3). These results directly correlate deubiquitination with the ability of Rad23 to deliver ubiquitinated substrates to the proteasome.

## Discussion

Interactions involving UBL and UBA domains are crucial to the function of the Rad23 family of proteins in ubiquitin-mediated protein degradation. In HHR23A, the intramolecular binding between the UBL and UBA domains is affected by and regulates interaction with other proteins [18]. We show that disruption of the intramolecular UBL-UBA binding facilitates HHR23A interaction with polyubiquitin. Our data support the model that binding to either proteasomes or polyubiquitin disrupts the UBL-UBA interaction, causing HHR23A to adopt an open conformation that facilitates its interaction with polyubiquitinated proteins or proteasomes respectively. This coupling of proteasome-binding and substrate-binding renders the Rad23 family more efficient in docking ubiquitinated substrates with the proteasome. Indeed, ubiquitin chains have been shown to enhance Rad23 binding to the proteasome *in vivo *[32]. Also, UBL-UBA domain interactions may enhance specificity by blocking interactions that are weaker than the intramolecular UBL-UBA interaction.

The UBA-binding surface of the Rad23/HHR23A UBL domain also mediates binding to the proteasomal subunit S5a as well as to other proteins such as the ubiquitin ligase Ufd2. We had targeted residues on the edge of the UBA-binding surface in an attempt to minimize disrupting other interactions but our mutations also affected proteasome-binding and Ufd2-binding. Our results are consistent with a previous report suggesting that the binding surfaces on the UBL domain for Ufd2 and the proteasome may overlap [28]. The difficulty of specifically disrupting interaction with only one protein without affecting others suggests that these different binding surfaces overlap significantly on the UBL domain and that the various interactions may regulate one another. The use of the same surface on Rad23/HHR23A for both proteasome- and Ufd2-binding is consistent with a model in which Rad23 first binds ubiquitin conjugates associated with Ufd2, then dissociates from Ufd2 and binds the proteasome, to which the ubiquitin conjugates are thus delivered. This model contrasts with one in which Rad23 bridges Ufd2 and the proteasome by binding them simultaneously.

The UBL mutants tested constitute an allelic series, with the S73E mutant behaving most similarly to the wild-type UBL, following by K43E and then F9E. Despite the lack of detectable proteasome-binding in the native gel assay, which is based on fractionation of the interacting species, the UBL mutants possess sufficient residual affinity that they can still interact functionally with proteasomes, as evidenced by the deubiquitination of Sic1. Rad23ΔUBL does not interact with proteasomes, yet it inhibited deubiquitination of Sic1. This dominant negative effect arises from a sequestering mechanism, as Rad23ΔUBL can still bind ubiquitin chains and thus interfere with recognition by intrinsic ubiquitin receptors and other shuttling factors. In contrast, Sic1 was deubiquitinated in the presence of the UBL mutants in a manner that is consistent with each mutant's relative ability to bind proteasomes. These data indicate that the capacity of Rad23 to bind proteasomes is essential for the ability of Rad23 family proteins to target ubiquitinated substrates to the proteasome. This model contrasts one in which ubiquitin chains bind the proteasome directly.

Interestingly, HHR23A does not dimerize [18] whereas Rad23 does so in a manner that involves the C-terminal UBA domain [33,34]. Rad23 can also heterodimerize with other UBL-UBA proteins such as Ddi1 [33,34] while HHR23A interacts with hPLIC2 [35]. Therefore, intermolecular UBL-UBA domain interactions may also play a regulatory role in the recruitment of ubiquitinated substrates to the proteasome.

## Conclusion

In conclusion, both the intramolecular as well as intermolecular interactions of the UBL and UBA domains are important for the function of Rad23 family proteins in the ubiquitin-proteasome pathway. In this study we have shown that physical interactions between the UBL and UBA domains couple proteasome binding via the UBL domain with ubiquitin-binding via the UBA domain, thus priming Rad23/HHR23A for its role in shuttling ubiquitinated substrates to the proteasome.

## Methods

### Plasmids

For bacterial expression, proteins were cloned into either pGEX-6p-1 (Pharmacia) or pET-23a (Novagen). Where applicable, the FLAG tag was inserted by QuikChange-XL mutagenesis (Stratagene). We used pGEX2TK-based plasmids to express Rad23 and Rad23ΔUBL proteins for radiolabelling [7]. HHR23A (GenBank:P54725), HHR23A UBL mutants, HHR23B (GenBank:P54727), and Rad23 (GenBank:P32628) UBL mutants were cloned into "pGEX-6pK," in which the heart muscle kinase recognition site present in pGEX-2TK was inserted upstream of the pGEX-6p-1 multiple cloning site by QuikChange-XL mutagenesis (Stratagene). The GST-Ufd2-myc plasmid has been previously described [28].

### Protein expression and purification

Protein expression was induced in BL21(DE3) bacteria with 0.4 mM isopropyl β-D-thiogalactoside (IPTG) for 4 hours at 37°C. For GST-fusion protein purification, bacteria were lysed by sonication in PBS containing 2 mM DTT, protease inhibitor cocktail (Roche), and 1% Triton X-100. The proteins were purified on glutathione-sepharose beads and either eluted with 10 mM reduced glutathione in 50 mM Tris-HCl pH 8.0 or cleaved from the resin-bound GST with PreScission protease (pGEX-6p1 proteins) or thrombin (pGEX-2TK proteins) according to the manufacturer's instructions. For the purification of polyhistidine-tagged proteins, the bacteria were lysed in 25 mM sodium phosphate pH 8, 300 mM NaCl, 10 mM imidazole, 2 mM DTT and protease inhibitor cocktail (Roche). The polyhistidine-tagged proteins were bound to Ni-NTA resin (QIAGEN), washed with 50 mM sodium phosphate pH 6.0, 300 mM NaCl and 20 mM imidazole. The proteins were then eluted with 25 mM Tris-HCl pH 8, 300 mM NaCl, 2 mM DTT and 0.25 M imidazole. Purified proteins were dialyzed overnight against PBS, 10% glycerol and 2 mM DTT. Protein purity was verified by SDS-PAGE analysis and Coomassie Blue staining.

Free polyubiquitin chains were synthesized *in vitro *by incubating 100 nM E1 (Boston Biochem), 10 μM E2-25K (Boston Biochem), and 40 μg ubiquitin (Sigma) with 25 mM Tris-HCl pH 7.6, 50 mM NaCl, 0.1 mM DTT, 5 mM MgCl_2_, 4 mM ATP (Sigma), 0.03 mg/ml creatine kinase, 5 mM creatine phosphate, and 0.3 unit/ml pyrophosphatase, at 30°C for 2 hours.

### Affinity column chromatography

Glutathione sepharose was saturated with purified GST-HHR23A~ΔUBL. The charged resin was then loaded into a 10 ml HR10/10 (Pharmacia) column. 1 μg each of wild-type UBL-His and mutated UBL-FLAG were mixed and loaded onto the GST-HHR23A~ΔUBL column, following which the column was developed in PBS. 0.5 ml fractions were collected, resolved by SDS-PAGE and the proteins were detected by Western blotting. Antibodies used included anti-FLAG M2 monoclonal antibody (Sigma), anti-HA 12CA5 monoclonal antibody (produced in our lab), and anti-polyhistidine affinity-purified rabbit polyclonal antibody (Rockland). Use of the Odyssey Imaging System (LI-COR Biotechnology) enabled each protein to be visualized simultaneously but distinctly.

### Competition assay to assess polyubiquitin-binding

GST-HHR23A, untagged mutant variants of GST-HHR23A, free polyubiquitin chains and glutathione-sepharose beads were mixed for 1–2 hours at 4°C in binding buffer (20 mM Tris-HCl pH 7.2, 150 mM NaCl, 2 mM EDTA, 2% (v/v) glycerol, 1 mM DTT) [14] supplemented with 1% BSA. The resin was washed with binding buffer supplemented with 0.5% NP40. Proteins were eluted from the glutathione-sepharose resin and analyzed by Western blotting for polyubiquitin (FK2 monoclonal antibody, Affiniti Research Products). The band corresponding to tetra-ubiquitin was quantified with a Biorad Fluor-S Max phosphoimager. Total binding was defined as the amount of polyubiquitin bound to the GST-HHR23A in the absence of competitor.

### Native gel assays for proteasome-binding

Proteasomes were purified from *S. cerevisiae *as previously described [7]. All Rad23/HHR23 proteins were bacterially expressed, purified on glutathione-sepharose beads, and labelled *in vitro *with [γ-^32^P]ATP (NEN) and heart muscle kinase according to the manufacturer's instructions (Pharmacia Biotech). The radiolabelled proteins were cleaved from the resin with thrombin or PreScission protease, then quantified by scintillation counting and by Bradford assay. The specific activities of the proteins were normalized and mixed with proteasomes in a 1:50, 1:20, and 1:10 molar excess of proteasome over proteins. After incubation at 30°C for 15 minutes, the mixtures were resolved by 3.5% native PAGE essentially as previously described [7,36], though electrophoresis was carried out for 5 hours and the proteins visualized by autoradiography.

### GST-pulldown assay for Ufd2-binding

Glutathione sepharose was saturated with purified GST-Ufd2. Purified Rad23 proteins were then added together with 50 mM HEPES pH 7.5, 150 mM NaCl, 5 mM EDTA, 2% Triton X-100, 0.2 mg/ml BSA, and protease inhibitor cocktail (Roche). After mixing at 4°C for 60 minutes, the resin was washed with PBS and 0.1% Tween-20. The resin-bound Rad23 proteins were detected by Western blotting using an antibody against Rad23 (a gift from Kiran Madura).

#### In vitro deubiquitination assay

The *in vitro *deubiquitination assay was performed as previously described [5]. Essentially, proteasomes were purified from Rad23-deficient *S. cerevisiae *and preincubated with 100 μM epoxomicin for 45 minutes at 30°C to inhibit the protease activity of the proteasome. Ubiquitinated MbpSic1 was then added to the proteasome and its deubiquitination was analyzed by Western blotting for Sic1. Rad23 was added to the deubiquitination assay where indicated.

## Abbreviations

UBL, ubiquitin-like; UBA, ubiquitin-associated; His, polyhistidine; Ub, ubiquitin; HHR23, Human Homolog of Rad23; *S. cerevisiae, Saccharomyces cerevisiae*.

## Authors' contributions

AMG participated in the design of the study, carried out experiments and drafted the manuscript. KJW created the models of the UBL domains, participated in the design of the study and helped to revise the manuscript. SE participated in the design of the study, contributed reagents, and helped to revise the manuscript. RV performed the *in vitro *deubiquitination studies and helped to revise the manuscript. RJD and DF participated in the design of the study and helped to revise the manuscript. PMH participated in the design and coordination of the study and helped to revise the manuscript. All authors read and approved the final manuscript.

## Supplementary Material

Additional file 1 **Retention of proteins on the GST-HHR23A~ΔUBL column corresponds to their ability to interact with UBA domains**. (A) Purified GST-HHR23A~ΔUBL protein was used to saturate glutathione-sepharose resin, which was in turn used to pack an HR10/10 column. Equal amounts of HA-tagged SUMO (HA-SUMO), polyhistidine-tagged ubiquitin (Ub-His) and FLAG-tagged UBL (UBL-FLAG) were loaded simultaneously onto the column and the column was resolved in PBS. Fractions were collected and analyzed by Western blotting using epitope-specific antibodies. (B) Purified GST was bound to glutathione-sepharose resin in excess, which was then used to pack an HR10/10 column. Equal amounts of HA-SUMO, Ub-His and UBL-FLAG were mixed, loaded onto the column and analyzed as described above. Click here for file
