# Supplementary material for: Components of the ubiquitin-proteasome pathway compete for surfaces on Rad23 family proteins
Source: BMC Biochem. 2008 Jan 30;9:4. doi: 10.1186/1471-2091-9-4 (PMC2267792; doi:10.1186/1471-2091-9-4)
Supplement: Additional file 1 — Retention of proteins on the GST-HHR23A~ΔUBL column corresponds to their ability to interact with UBA domains. (A) Purified GST-HHR23A~ΔUBL protein was used to saturate glutathione-sepharose resin, which was in turn used to pack an HR10/10 column. Equal amounts of HA-tagged SUMO (HA-SUMO), polyhistidine-tagged ubiquitin (Ub-His) and FLAG-tagged UBL (UBL-FLAG) were loaded simultaneously onto the column and the column was resolved in PBS. Fractions were collected and analyzed by Western blotting using epitope-specific antibodies. (B) Purified GST was bound to glutathione-sepharose resin in excess, which was then used to pack an HR10/10 column. Equal amounts of HA-SUMO, Ub-His and UBL-FLAG were mixed, loaded onto the column and analyzed as described above. [file 1471-2091-9-4-S1.PPT]

## Slide 1
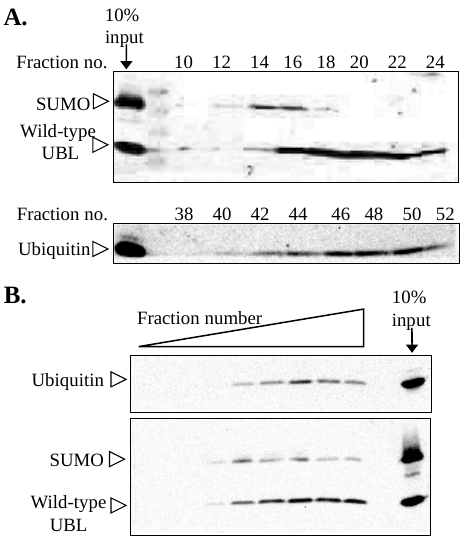

A.
10%
input
Fraction no. 10 12 14 16 18 20 22 24
SUMO
Wild-type
UBL
Fraction no. 38 40 42 44 46 48 50 52
Ubiquitin
B.
10%
input
Fraction number
Ubiquitin
SUMO
Wild-type
UBL
